# Necrotizing scleritis as a complication of cosmetic eye whitening procedure

**DOI:** 10.1186/1869-5760-3-39

**Published:** 2013-02-22

**Authors:** Theresa G Leung, James P Dunn, Esen K Akpek, Jennifer E Thorne

**Affiliations:** 1 The Division of Ocular Immunology, The Wilmer Eye Institute, Johns Hopkins School of Medicine, 600 North Wolfe Street, Woods Building, Room 476, Baltimore, MD 21287, USA; 2 The Division of Corneal and External Diseases, The Wilmer Eye Institute, Johns Hopkins School of Medicine, Baltimore, MD 21287, USA; 3 Department of Epidemiology, Johns Hopkins University Bloomberg School of Public Health, Baltimore, MD 21287, USA; 4 The Wilmer Eye Institute, Johns Hopkins School of Medicine, 600 North Wolfe Street, Woods Building, Room 476, Baltimore, MD 21287, USA

**Keywords:** Necrotizing scleritis, I-BRITE, Cosmetic eye whitening, Mitomycin C

## Abstract

**Background:**

We report necrotizing scleritis as a serious complication of a cosmetic eye whitening procedure that involves the use of intraoperative and postoperative topical mitomycin C.

**Findings:**

This is a single case report. A 59-year-old Caucasian male with a history of blepharitis status post uncomplicated LASIK refractive surgery reported chronic conjunctival hyperemia for 15 years prior to undergoing a cosmetic eye whitening procedure. He presented to our clinic 12 months after the cosmetic eye whitening procedure with progressive bilateral necrotizing scleritis and scleral calcification.

**Conclusions:**

Chronic conjunctival hyperemia may prompt patients to seek surgical correction with cosmetic eye whitening procedures. However, conjunctival hyperemia secondary to tear deficiency and evaporative dry eye may predispose to poor wound healing. Serious complications including necrotizing scleritis may result from cosmetic eye whitening procedures and the use of topical mitomycin C.

## Findings

### Introduction

Cosmetic eye whitening procedures are a relatively new cosmetic treatment option for patients concerned about the appearance of chronic conjunctival hyperemia. There are a limited number of centers in the USA and worldwide that perform eye whitening procedures. The eye whitening procedure is similar to the conjunctivoplasty performed in pterygium excision but involves dissection and cautery of the conjunctiva tissue down to the level of episclera and portions of Tenon’s capsule with intraoperative or postoperative use of topical mitomycin C [1,2]. The out-of-pocket expense for the procedure is approximately US$3,000 to 5,500 per eye [1].

Kim reported a technique of regional conjunctivectomy with postoperative mitomycin C 0.02%, known commercially as Cosmetic Eye Whitening™ (Seer & Partner Eye Institute, Inc., Seoul, South Korea) [3]. Postoperative management includes topical application of 0.02% mitomycin C four times daily for 2 to 5 days, antibiotic eye drops, and topical corticosteroid eye drops [3]. The desired outcome is for regenerated conjunctival tissue to appear translucent without the excessive vascularization that causes the appearance of red eyes.

Serious complications related to the use of mitomycin C in eye whitening procedures have been reported in patients undergoing the Cosmetic Eye Whitening™ procedure, including avascularity of the sclera, chronic conjunctival epithelial defects, scleral thinning with or without calcified plaques, adhesions of Tenon’s capsule to the conjunctiva at the extraocular muscle insertion site, extraocular muscle fiber exposure, and diplopia [4].

We report a case of bilateral necrotizing scleritis after the I-BRITE™ cosmetic eye whitening procedure performed in the USA. To our knowledge, this represents the first documented case of a bilateral necrotizing scleritis as a complication of an eye whitening procedure.

### Case presentation

A 59-year-old Caucasian male presented to our clinic in February of 2011 for a second opinion regarding the development of white spots that he noticed in the inner corner of both eyes. His past medical history was notable only for hay fever. He had uncomplicated LASIK refractive surgery in both eyes in 1995. He reported a 15-year history of chronic red eyes and irritation which prompted him to undergo the I-BRITE™ (Boxer Wachler Vision Institute, Beverly Hills, CA, USA) cosmetic eye whitening surgery in both eyes in February 2010.

Initially after the eye whitening procedure, he noticed decreased redness in both eyes. Four to five months following the procedure, however, he noted ‘white spots’ appearing in the inner corner of both eyes. There was no associated pain, discharge, vision change, or sensitivity to light. He was treated by his local ophthalmologist with cyclosporine 0.05%, olopatadine 0.1%, epinastine 0.05%, and punctal plugs in the lower lids bilaterally without improvement of the white spots.

**Figure 1:**
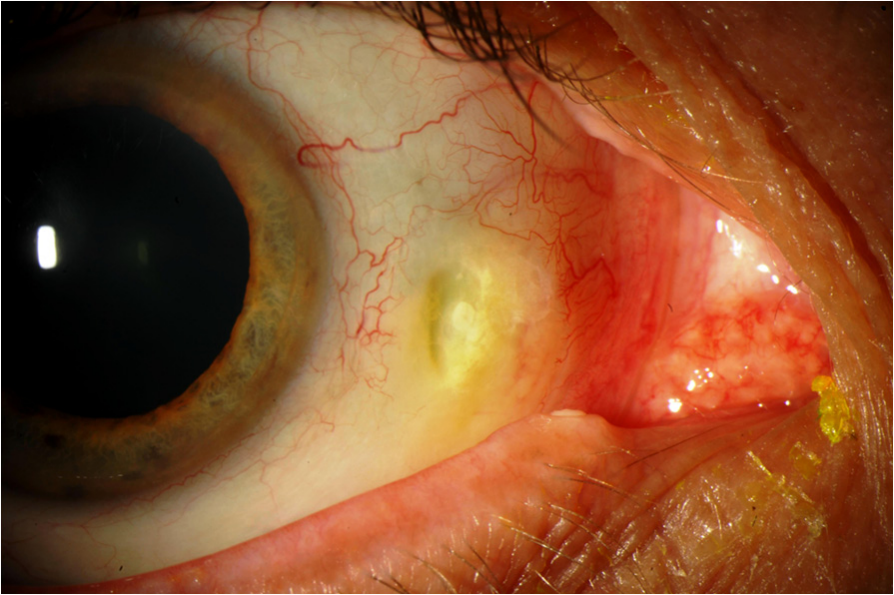
Necrotizing scleritis with associated calcium plaque, right eye.

**Figure 2:**
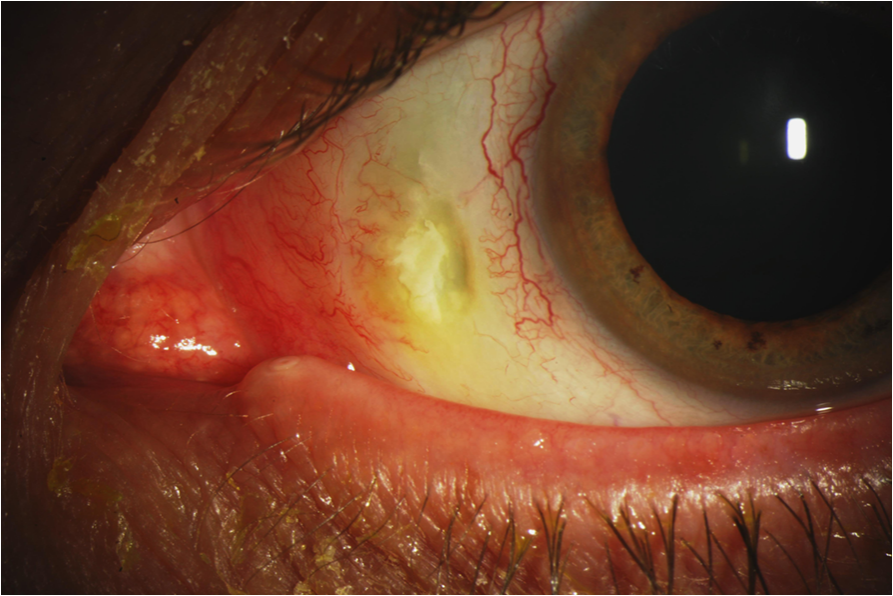
Necrotizing scleritis with associated calcium plaque, left eye.

**Figure 3:**
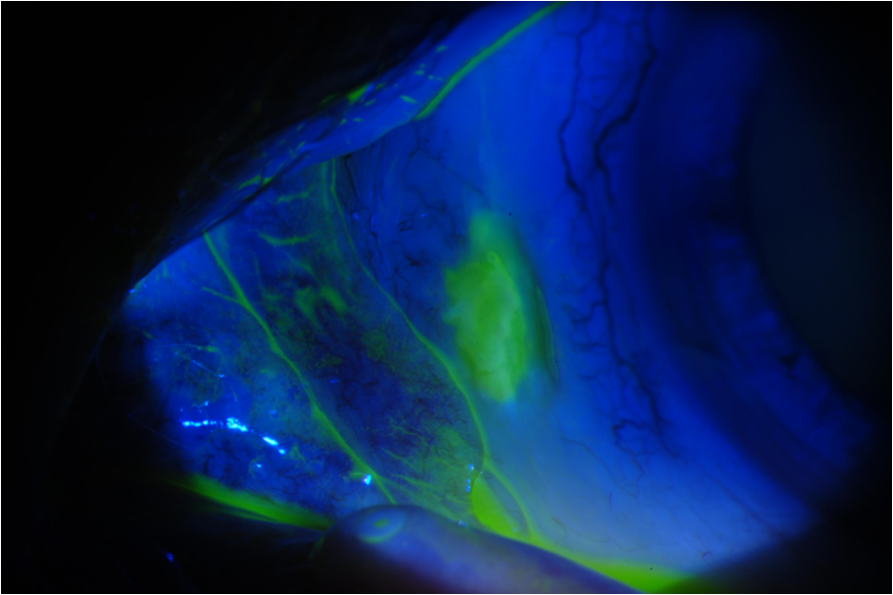
Fluorescein uptake in the area of conjunctival and sclera erosion.

At the time of presentation, he was using ketorolac 0.5% and artificial tears in both eyes three times daily. Uncorrected visual acuity at distance was 20/20 in both eyes. Slit-lamp examination of the lids and lashes revealed meibomian gland dysfunction with telangiectactic vessels along the lid margins. Zones of markedly white conjunctivae and sclerae were noted bilaterally. In the right medial conjunctiva, there was a 2 × 1 mm region of conjunctival erosion with fluorescein uptake associated with scleral thinning and calcium deposits (Figure 1). A prominent finding was the occurrence of the scleral thinning in a region lacking conjunctival, episcleral, or scleral vessels. Similarly, the medial conjunctiva of the left eye demonstrated a 2.5 × 1 mm region of conjunctival erosion and scleral melting, associated with calcium deposits (Figures 2 and 3). There were well-healed LASIK scars bilaterally, and there was no anterior chamber reaction.

Aggressive lubrication with preservative-free artificial tears and ointment was initiated, and the ketorolac was discontinued. Over the next 4 months, there was evidence of progressive scleral melting in both eyes. At that point, an amniotic membrane patch graft was recommended. However, the patient was reluctant to undergo further surgical procedures and elected alternative medical management with cyclosporine 1% and medroxyprogesterone acetate 1% eye drops. He has been maintained on these eye drops with close monitoring.

### Discussion

Necrotizing scleritis with or without inflammation has been reported as a complication of pterygium excision with adjunctive mitomycin C therapy [5-9]. Other documented complications from the intraoperative and postoperative use of topical mitomycin C include scleral ulceration, scleral calcification, scleral thinning, chemosis, delayed conjunctival wound healing, corneoscleral melt, corneal edema, keratitis, corneal perforation, secondary glaucoma, corectopia, and uveitis [5-11].

Mitomycin C is an alkylating agent that inhibits DNA synthesis. The mechanism behind necrotizing scleritis and sclera thinning secondary to mitomycin C is thought to be inhibition of fibroblast proliferation coupled with vascular compromise leading to an avascular necrosis of the sclera [7,12]. One study examined the scleral thickness and the conjunctival epithelium after pterygium excision with intraoperative mitomycin C. After a mean period of 6 years, there was a fourfold decrease in the goblet cell density of the surgical site after mitomycin C application compared to the contralateral non-operated site [12]. This demonstrates that mitomycin C has prolonged effects on the ocular surface tissue long after discontinuation of the drug.

Cosmetic eye whitening procedures, similar to pterygium excision, involve wide conjunctivectomy and intraoperative and/or postsurgical or topical application of 0.02% mitomycin. In a retrospective study conducted by Rhiu et al. [4], complications were found in 44 of 48 patients (92%), including 22 patients (50%) with chronic conjunctival epithelial defects, 21 (48%) with scleral thinning, 19 (43%) with calcified plaques, and 10 (23%) with avascular zones of the sclera. It is possible that these ten patients with avascular zones may have presented with the same clinical phenomenon as our patient. Conversely, Kim [3] reported a much lower incidence of complications, including scleral calcification (3.9%), diplopia (1.6%), elevated intraocular pressure secondary to postoperative topical steroid use (17.2%), granuloma formation (8.4%), and transient decrease in vision (7.5%) without permanent deficits noted.

In our patient, the necrotizing scleritis presented with bilateral focal scleral thinning and calcified plaques. We believe that the vascular compromise precipitated the necrotizing scleritis as there was a marked scarcity of conjunctival and sclera vessels in the affected region.

Treatment options for necrotizing scleritis secondary to pterygium excision with mitomycin C include a scleral patch, amniotic membrane patch graft, lamellar corneoscleral graft, or conjunctival flap [13-15]. Five patients in the study conducted by Rhiu et al. required a conjunctival flap because of severe progressive scleral thinning [4]. Our patient declined an amniotic membrane patch graft after a discussion of the possible failure of a conjunctival flap in an eye with compromised vasculature.

In cases of pterygium excision, Rubinfeld et al. [6] cautioned that conditions such as acne rosacea, atopic keratoconjunctivitis, keratoconjunctivitis sicca, Sjögren’s syndrome, blepharitis, or herpes keratitis may predispose to poor wound healing, making mitomycin C a precarious choice of therapy in affected patients. Unfortunately, these are many of the same conditions that cause chronic conjunctival injection prompting patients to seek cosmetic eye whitening procedures.

Our patient had prior LASIK surgery and blepharitis, both of which can cause or exacerbate dry eye and may have been the underlying cause of his chronic conjunctival injection before he sought a surgical cosmetic solution. Tear deficiency and evaporative dry eye may have predisposed him to poor wound healing, in addition to the postoperative use of topical NSAIDs.

Although the technique developed by Kim in his Cosmetic Eye Whitening™ procedure has been published and reported, we acknowledge there may be slight variations from the I-BRITE™ procedure that our patient underwent which have not been published in the literature [3], such as the concentration or application time of intraoperative mitomycin C and the concentration, duration, and frequency of the mitomycin C postoperatively. These variations may account for why more reported complications have not arisen in the USA from patients who have undergone cosmetic eye whitening, or it may be that the number of patients who have had this procedure in the USA has not reached a significant level in comparison to South Korea.

While elective cosmetic eye whitening procedures may be safe in most patients, our case reaffirms that serious complications may occur with surgical and pharmacologic manipulation of the natural vasculature of the ocular surface.

### Consent

Written informed consent was obtained from the patient for publication of this report and any accompanying images.

## Competing interest

The authors have no financial or other competing interests in the products or materials published in this report.

## Authors’ contributions

TGL, JPD, EKA, and JET participated in the drafting of the manuscript. TGL, JPD, and EKA participated in the management of the patient. All authors read and approved the final manuscript.
